# Association of chronic insomnia symptoms and recurrent extreme sleep duration over 10 years with well-being in older adults: a cohort study

**DOI:** 10.1136/bmjopen-2015-009501

**Published:** 2016-02-01

**Authors:** Jessica G Abell, Martin J Shipley, Jane E Ferrie, Mika Kivimäki, Meena Kumari

**Affiliations:** 1 Department of Epidemiology and Public Health, University College London, London, UK; 2 School of Social and Community Medicine, University of Bristol, Bristol, UK; 3 Institute for Social and Economic Research, University of Essex, Colchester, UK

**Keywords:** EPIDEMIOLOGY, MENTAL HEALTH, PUBLIC HEALTH, SOCIAL MEDICINE

## Abstract

**Objectives:**

The extent to which aspects of sleep affect well-being in the long-term remains unclear. This longitudinal study examines the association between chronic insomnia symptoms, recurrent sleep duration and well-being at older ages.

**Setting:**

A prospective cohort of UK civil servants (the Whitehall II study).

**Participants:**

4491 women and men (25.2% women) with sleep measured 3 times over 10 years and well-being once at age 55–79 years. Insomnia symptoms and sleep duration were assessed through self-reports in 1997–1999, 2003–2004 and 2007–2009.

**Primary outcome measures:**

Indicators of well-being, measured in 2007–2009, were the Control, Autonomy, Self-realisation and Pleasure measure (CASP-19) of overall well-being (range 0–57) and the physical and mental well-being component scores (range 0–100) of the Short Form Health Survey (SF-36).

**Results:**

In maximally adjusted analyses, chronic insomnia symptoms were associated with poorer overall well-being (difference between insomnia at 3 assessments vs none −7.0 (SE=0.4) p<0.001), mental well-being (difference −6.9 (SE=0.4), p<0.001) and physical well-being (difference −2.8 (SE=0.4), p<0.001) independently of the other sleep measures. There was a suggestion of a dose–response pattern in these associations. In addition, recurrent short sleep (difference between ≤5 h sleep reported at 3 assessments vs none −1.7 (SE=0.7), p<0.05) and recurrent long sleep (difference between >9 h reported at 2 or 3 assessments vs none −3.5 (SE=0.9), p<0.001) were associated with poorer physical well-being.

**Conclusions:**

We conclude that in older people, chronic insomnia symptoms are negatively associated with all aspects of well-being, whereas recurrent long and short sleep is only associated with reduced physical well-being.

**Strengths and limitations of this study**

- So far most evidence on the association between quality sleep and well-being has been drawn from cross-sectional data and has focused on health-related well-being measures.
- Strengths of this study include the availability of repeat measures of sleep duration and insomnia symptoms and three validated well-being scales to consider different domains of well-being.
- It suggests that there are long-term effects of insomnia symptoms for the well-being of older people. However, negative effects of extreme sleep duration are only seen for physical well-being.
- A limitation of this study is that these sleep measures are self-reported. Although observational are beginning to utilise actigraphy methods, these were not available over such a long time period.

## Introduction

Insomnia symptoms, short (≤5 h/night) and long (≥9 h/night) sleep are all associated with an increased risk of a range of chronic health conditions, such as diabetes,^1–3^ hypertension^4^ and mortality.^5^ ^6^ Health is an important predictor of well-being; however, overall well-being is often more than merely the absence of poor physical or mental ill health. This is particularly the case in older populations, where there is a high prevalence of chronic diseases.

Cross-sectional research on the contribution of sleep to well-being indicates that insomnia symptoms^7–9^ and both short and long sleep^10–12^ are associated with lower levels of well-being. Evidence for an interaction between insomnia symptoms, sleep duration and health has also been suggested.^13^ However, what has been studied less is whether these cross-sectional associations strengthen when insomnia symptoms and extreme sleep duration are based on repeated assessments. A recent study measured chronic insomnia symptoms at two time points, using a conservative estimate; the lowest frequency of insomnia symptoms mentioned at either of the time points.^8^ The study found that these had a strong negative association with subjective well-being.

The relationship between sleep and well-being might also vary with the outcome measure examined. In previous work there has been an emphasis on measures which capture health-related well-being, such as the Short Form (SF-36) Health Survey.^14^ However, this may not fully capture well-being in elderly populations, since it reflects mental and physical functioning which decline in older age groups.^15^ To evaluate overall well-being in early old age, the Control, Autonomy, Self-realisation and Pleasure (CASP-19) measure was developed. It evaluates quality of life as distinct from factors which predict it, such as good health.^16^

To the best of our knowledge, no other studies have been able to provide repeat measurements taken over a 10-year follow-up period. To address these limitations of previous work, we examine reports of chronic insomnia symptoms and recurrent extreme sleep duration with well-being in old age. Our two key objectives are: (1) to examine whether chronic insomnia symptoms and recurrent short or long sleep duration are independently associated with well-being in older adults and (2) to determine whether the associations between sleep and well-being extend to three different domains: overall well-being (CASP-19), physical well-being (SF-36: PCS) and mental well-being (SF-36: MCS).

## Methods

### Study sample

The Whitehall II Cohort was recruited from London-based Civil Service departments in 1985–1988 (phase 1), the sample consisted of 10 308 participants aged 35–55, with a response rate of 73%. Follow-up screening examinations took place in 1991–1993 (phase 3) and 1997–1999 (phase 5), 2003–2004 (phase 7) and 2007–2009 (phase 9) with postal questionnaires being sent to participants in 1989 (phase 2), 1995 (phase 4), 2001 (phase 6) and 2006 (phase 8). Further details of the Whitehall II Study can be found elsewhere.^17^ In this study, we used sleep exposure data from 1997–1999, 2003–2004 and 2007–2009 to predict well-being in 2007–2009, when the participants were aged 55–79 years. A total of 6761 respondents participated in phase 9, a response rate of 66% since phase 1, but 86% from those eligible at phase 9. The follow-up rate from phases 5 to 9 was 85.9%. The final sample of 4491 (1133 women; 25.2%) participated at phase 9 and had complete information for all relevant variables.

### Well-being outcomes

The following outcome measures reported at phase 9 (2007–2009) were used in the analysis:

- *Overall well-being (CASP-19)*: CASP-19 is an instrument developed and validated to measure overall well-being in older people, independent of influencing factors such as health.^18^ CASP-19 sums 19 Likert-scaled items, measuring Control, Autonomy, Self-realisation and Pleasure. Testing carried out on CASP-19 during its development is reported elsewhere.^19^ Respondents were asked to indicate how often each statement applied to them; *often, sometimes, not often or never*, and these scores were appropriately coded, using a sliding scale of 0–3 and summed (range 0–57), with higher scores indicating a better quality of life.^19^ ^20^ The scale had good internal consistency at phase 9 (2007–2009; Cronbach's α=0.88).
- *Physical and mental well-being (SF-36)*: SF-36 is a 36-item questionnaire; these questions are used to construct the eight SF-36 scales: physical functioning, mental functioning, role limitations due to physical problems, social functioning, bodily pain, role limitations due to emotional problems, vitality and general health perceptions.^21^ These eight scales can be aggregated to form two summary scores—physical and mental functioning component scores—using a method based on factor analysis. They are considered to be conceptually distinct measures of physical (SF-36: PCS) and mental well-being (SF-36: MCS).^14^ ^21^ Scores for each of these two scales ranged from 0 to 100, with higher scores indicating greater well-being. The correlation between CASP-19 and SF-36 mental well-being was r=0.64 (p≤0.001) and the correlation between CASP-19 and SF-36 physical well-being was r=0.39 (p≤0.001).

### Measures of sleep

*Insomnia symptoms* were measured at the same phases as sleep duration using the Jenkins’ sleep problem scale.^22^ Participants were asked how many times during the last month they: (1) ‘have trouble falling asleep’, (2) ‘have trouble staying asleep (ie, waking up far too early)’, (3) ‘wake up several times per night’ and (4) ‘wake up after usual amount of sleep feeling tired and worn out’. The following response categories were available: *not at all, 1–3, 4–7, 8–14, 15–21 and 22–31 days*. This scale was summed and grouped into quartiles. The first three quartiles were grouped together (low insomnia symptoms) and the fourth quartile was grouped separately (high insomnia symptoms). Chronic insomnia symptoms were defined as the number of times, across the three time points that a participant reported high insomnia symptoms. The length of follow-up from the first sleep exposure to outcome ranged from 8 to 12 years (mean 9.8 years).

*Sleep duration* was self-reported and measured at phase 5 (1997–1999), phase 7 (2003–2004) and phase 9 (2007–2009) using the question: ‘How many hours of sleep do you have on an average week night?’; with the options *5 h or less, 6, 7, 8* or *9 h or more*. Cross-sectional research (see online supplementary table S1) confirmed evidence from previous literature, that extreme sleep duration has the greatest impact on health and well-being, therefore only short and long sleep was examined longitudinally. Two variables were created using data from each time point: (1) recurrent short sleep, defined as the number of times a participant reported short (≤5 h/night) sleep across the three time points; (2) recurrent long sleep, defined as the number of times a participant reported long sleep (≥9 h/night) across the three time points.

### Covariates

A range of covariates, measured at phase 9 (2007–2009), were also included: *gender* and *age* were considered to be confounding factors. A quadratic term for age (age^2^) was included because the relationship of age to CASP-19 has been shown to follow a non-linear trend.^16^ Participants were asked to estimate their total *household wealth* (including house value); this was recoded into four categories (1) <£200 000, (2) £200–£499 999, (3) £500–£999 999 and (4) >£1 000 000. Household wealth rather than civil service employment grade or income was used since it has been shown to represent the economic status of older people more accurately than income.^23^ A binary variable indicated whether the participant was still in paid *employment. Marital status* was defined as married/cohabiting or not. *Chronic health conditions* were assessed as the presence or absence of a limiting long-term illness. *Poor functioning* was defined as limitations in one or more activities of daily living (ADL), or one or more instrumental ADL (IADL). *Health behaviours*: smoking (current vs never/ex-smokers), physical activity; based on the duration of ‘vigorous’ activity (≥1.5 vs <1.5 h per week). Physical activity was assessed using a questionnaire which asked participants about the number of hours spent undertaking a range of physical activity (both leisure time and job-related activities). Each activity was assigned a metabolic equivalent (MET) value.^24^ Vigorous physical activity was defined as activities with a MET value of 6 or more^25^ (eg, swimming, mowing). High alcohol consumption (≥14 units/week for women and ≥22 units/week for men) and body mass index (BMI): height and weight were measured during the medical examination and BMI (kg/m^2^) calculated. *Depressive symptoms* were assessed using a modified version of the 30-item General Health Questionnaire (GHQ)^26^ removing the two questions that referred to sleep problems. Higher GHQ scores indicate more depressive symptoms.

### Statistical analysis

Pearson’s χ^2^ test for homogeneity (4df) was used to examine this association between sleep duration and each categorical covariate, while linear regression was used for continuous exposures to examine heterogeneity across the sleep duration categories. We also conducted a non-parametric test of trend for each well-being outcome, across the groups of each exposure variable. We used the Stata command *nptrend* which is an extension of the Wilcoxon rank-sum test. Three models were estimated using the exposures for recurrent short and long sleep and chronic insomnia symptoms. In the first model age, age^2^, gender and household wealth were included. In model 2 employment status, marital status, chronic health conditions, ADL/IADL and health behaviours were additionally included. In model 3, the remaining sleep exposure was also added to model 2. Since the association between overall well-being, or physical well-being and poor sleep might be confounded by mental health, further models were adjusted for the depressive symptoms score. Statistical significance levels were set at p<0.05 for two-sided analyses. Each exposure variable was also examined cross-sectionally; these results are available in online supplementary tables S1 and S2 and the results reported in the text. In the cross-sectional analysis, the full five-category measure of sleep duration was tested and each item of the insomnia symptoms scale examined separately. In the cross-sectional models, a reference group of 7 h was used.^27^ All analyses were undertaken using Stata V.13.1.

## Results

The distribution of participant characteristics, by sleep duration reported in 2007–2009 is reported in table 1. In this sample, the mean (SD) overall well-being score was 43.5 (7.8), the mean physical well-being score was 49.0 (8.5) and the mean mental well-being score was 53.9 (7.9). The percentage of those participants who reported high levels of insomnia symptoms at each of the three time points was 8.2% (N=368); in 2007–2009, 7.5% (N=335) participants reported short sleep and 2.1% (N=94) long sleep. An inverted U-shaped association with sleep duration was observed for each of these outcomes. Those who reported shorter and longer sleep were also more likely to have a long-term illness and have one or more ADLs and IADLs. Those who reported sleeping 5 h or less were more likely to be younger, female and to have worked or be currently working in the lowest civil service employment grade, but were less likely to be married or cohabiting. They were also more likely to have a high BMI, less likely to report undertaking any vigorous physical activity and more likely to score highly on the GHQ depression scale and report high levels of insomnia symptoms.

**Table 1:** Characteristics of participants by sleep duration 2007–2009 (N=4491)

	Hours of sleep						
	All	≤5	6	7	8	≥9	p Value*
Sleep duration, % (N)		7.5 (335)	29.0 (1303)	41.8 (1875)	19.7 (884)	2.1 (94)	
Age (years), mean (SD)†	65.6 (5.9)	66.5 (6.1)	65.5 (5.9)	65. 4 (5.8)	66.1 (5.7)	67.4 (6.2)	<0.0001
Women, % (N)	25.2 (1133)	36.4 (122)	26.9 (351)	24.6 (461)	20.1 (178)	22.3 (21)	<0.0001
Married, % (N)	76.8 (3449)	58.8 (197)	74.2 (967)	79.4 (1489)	81.8 (723)	77.7 (73)	<0.0001
Employed, % (N)	31.5 (1414)	28.7 (96)	36.9 (481)	34.1 (640)	20.9 (185)	12.8 (12)	<0.0001
Lowest wealth (<£200 000), % (N)	9.3 (419)	17.9 (60)	10.1 (132)	8.8 (164)	6.5 (57)	6.4 (6)	<0.0001
High alcohol consumption, % (N)	17.8 (800)	13.4 (45)	17.2 (224)	17.6 (330)	19.9 (176)	26.6 (25)	0.015
Vigorous physical activity, % (N)	13.3 (595)	9.3 (31)	12.0 (156)	13.4 (251)	16.4 (145)	12.8 (12)	0.007
Current smoking, % (N)	6.3 (283)	5.4 (18)	5.4 (70)	6.7 (125)	7.1 (63)	7.5 (7)	0.366
BMI (kg/m^2^), mean (SD)†	26.6 (4.3)	27.4 (4.5)	27.0 (4.6)	26.5 (4.2)	26.1 (4.0)	26.7 (4.6)	<0.0001
No long-term illness, % (N)	34.6 (1555)	24.5 (82)	33.5 (437)	35.9 (673)	37.6 (332)	33.0 (31)	<0.0001
One or more ADL % (N)	8.5 (382)	15.8 (53)	10.3 (134)	6.8 (128)	6.3 (56)	11.7 (11)	<0.0001
One or more IADL % (N)	12.4 (555)	21.8 (73)	14.4 (188)	10.2 (192)	9.3 (82)	21.3 (20)	<0.0001
GHQ (modified), mean (SD) †	1.9 (4.1)	4.0 (6.1)	2.3 (4.5)	1.5 (3.6)	1.3 (3.1)	2.0 (3.8)	<0.0001
High insomnia symptoms, % (N)	32.5 (1461)	64.5 (216)	37.2 (484)	27.1 (508)	25.0 (221)	34.0 (32)	<0.0001
Chronic insomnia symptoms, % (N)‡							
No occurrence	63.3 (2842)	26.0 (87)	53.0 (690)	70.9 (1329)	76.4 (675)	64.9 (61)	<0.0001
One occurrence	17.4 (782)	20.6 (69)	20.6 (269)	15.3 (286)	15.8 (140)	19.2 (18)	<0.0001
Two occurrences	11.1 (499)	22.4 (75)	15.4 (200)	9.4 (176)	4.9 (43)	5.3 (5)	<0.0001
Three occurrences	8.2 (368)	31.0 (104)	11.06 (144)	4.5 (84)	2.9 (26)	10.6 (10)	<0.0001
Trouble falling asleep, %(N)	3.1 (140)	20.0 (67)	3.3 (43)	1.1 (20)	1.0 (9)	1.1 (1)	<0.0001
Waking in the night, % (N)	28.4 (1275)	54.0 (181)	31.5 (411)	23.9 (448)	23.3 (206)	30.9 (29)	<0.0001
Waking up tired, % (N)	7.1 (317)	26.6 (89)	8.0 (104)	4.4 (83)	3.4 (30)	11.7 (11)	<0.0001
Trouble staying asleep, % (N)	13.1 (588)	52.2 (175)	18.9 (246)	6.8 (128)	3.7 (33)	6.4 (6)	<0.0001
CASP-19, mean (SD)†	43.5 (7.8)	38.7 (9.2)	42.4 (7.8)	44.4 (7.2)	45.0 (7.1)	42.8 (8.1)	<0.0001
SF-36 (PCS), mean (SD)†	49.0 (8.5)	45.5 (10.5)	48.4 (9.1)	49.7 (7.9)	49.8 (7.8)	46.1 (8.8)	<0.0001
SF-36 (MCS), mean (SD)†	53.9 (7.9)	50.0 (10.6)	53.2 (8.2)	54.5 (7.3)	55.0 (6.8)	53.7 (8.7)	<0.0001

*p Value for heterogeneity.

†Mean (SD).

‡Number of times (1997–1999, 2003–2004 and 2007–2009) high level of insomnia symptoms reported.

ADL, activities of daily living; BMI, body mass index; CASP-19, Control, Autonomy, Self-realisation and Pleasure measure; GHQ, General Health Questionnaire; IADL, instrumental activities of daily living; SF-36 (MCS), Short Form Health Survey mental well-being component scores; SF-36 (PCS), Short Form Health Survey physical component scores.

In the cross-sectional linear regression analyses (see online supplementary tables S1 and S2), the binary measure of high levels of insomnia symptoms was associated with lower levels of all the well-being measures in each of the models. These associations were attenuated when covariates were included, especially for the measure of physical well-being. Negative associations were also observed between each of the three outcome measures and each item of the Jenkins sleep scale, when these were included in the analysis individually. A negative association between short sleep (≤5 or 6 h) was observed for both mental well-being and overall well-being when compared with those who report sleeping 7 h a night. However, a strong U-shaped association was observed between sleep duration and physical well-being SF-36 (PCS) in all three models, with both short (≤5 h) and long (≥9) sleep being associated with worse physical well-being.

Table 2 shows the results for recurrent short sleep, recurrent long sleep and chronic insomnia symptoms with well-being. A test for trend showed a trend of each well-being outcome across the occurrence of insomnia symptoms (CASP-19, p≤0.001; SF-36 (PCS), p≤0.001; SF-36 (MCS), p≤0.001). When chronic insomnia symptoms were examined in regression analysis a dose–response association was observed for each well-being outcome, with each additional occurrence of high levels of insomnia symptoms increasing the negative effect. This association remained in all three models, although the association was attenuated in the fully adjusted model. In models 1 and 2 recurrent short sleep (≤5 h) was associated with poorer overall well-being, with a small dose–response relationship suggested. A test of trend analysis indicated a trend for each of the well-being outcomes across the occurrences of short sleep (CASP-19, p≤0.001; SF-36 (PCS), p≤0.001; SF-36 (MCS), p≤0.001). However, when chronic insomnia symptoms were also included in the analysis, this association was attenuated substantially.

**Table 2:** Association of recurrent sleep duration and insomnia symptoms with overall well-being, physical well-being and mental well-being

N=4,491	Overall well-being b†			Physical well-being‡			Mental well-being§		
	Model 1 Diff¶ (SE) [standardised difference]	Model 2 Diff¶ (SE) [standardised difference]	Model 3 Diff¶ (SE) [standardised difference]	Model 1 Diff¶ (SE) [standardised difference]	Model 2 Diff¶ (SE) [standardised difference]	Model 3 Diff¶ (SE) [standardised difference]	Model 1 Diff¶ (SE) [standardised difference]	Model 2 Diff¶ (SE) [standardised difference]	Model 3 Diff¶ (SE) [standardised difference]
Recurrent short sleep††									
No short sleep (N=3869)	0.00 REF	0.00 REF	0.00 REF	0.00 REF	0.00 REF	0.00 REF	0.00 REF	0.00 REF	0.00 REF
One occurrence (N=372)	−3.23 (0.41) [−0.11] ***	−2.61 (0.39) [−0.09] ***	−0.96 (0.38) [−0.03]**	−2.28 (0.44) [−0.07] ***	−0.75 (0.37) [−0.02] *	0.01 (0.37) [−0.00]	−2.56 (0.42) [−0.09] ***	−2.34 (0.41) [−0.08] ***	−0.69 (0.41) [−0.02]
Two occurrences (N=147)	−3.38 (0.63) [−0.08] ***	−2.76 (0.60) [−0.06] ***	−0.73 (0.58) [−0.02]	−2.64 (0.69) [−0.05] ***	−1.37 (0.57) [−0.03] **	−0.56 (0.57) [−0.01]	−1.91 (0.65) [−0.04] ***	−1.57 (0.64) [−0.04] **	0.43 (0.62) [−0.01]
Three occurrences (N=103)	−4.66 (0.75) [−0.09] ***	−3.80 (0.71 [−0.07)***]	−0.84 (0.70) [−0.01]	−4.59 (0.82) [−0.08] ***	−2.83 (0.67) [−0.05] ***	−1.68 (0.68) [−0.03] **	−3.03 (0.78) [−0.06] ***	−2.68 (0.76) [−0.05] ***	0.21 (0.75) [−0.00]
Recurrent long sleep ‡‡									
No long sleep (N=4302)	0.00 REF	0.00 REF	0.00 REF	0.00 REF	0.00 REF	0.00 REF	0.00 REF	0.00 REF	0.00 REF
One occurrence (N=134)	−1.86 (0.67) [−0.04] **	−0.97 (0.63) [−0.02]	−1.04 (0.60) [−0.02]	−2.61 (0.72) [−0.05] **	−0.67 (0.59) [−0.01]	−0.67 (0.58) [−0.01]	−1.77 (0.69) [−0.04] *	−1.36 (0.67) [−0.03] *	−1.41 (0.64) [−0.03] *
Two or three occurrences (N=55)	0.68 (1.03) [−0.01]	0.03 (0.97) [−0.00]	0.43 (0.92) [−0.01]	4.19 (1.11) [−0.05] ***	3.33 (0.91) [−0.04] ***	−3.52 (0.90) [−0.05] ***	−0.91 (1.06) [−0.01]	−0.38 (1.03) [−0.01]	−0.78 (0.99) [−0.01]
Chronic insomnia symptoms§§									
No insomnia symptoms (N=2842)	0.00 REF	0.00 REF	0.00 REF	0.00 REF	0.00 REF	0.00 REF	0.00 REF	0.00 REF	0.00 REF
One occurrence (N=782)	−3.22 (0.29) [−0.16] ***	−2.83 (0.28) [−0.14] ***	−2.72 (0.28) [−0.13] ***	−2.74 (0.32) [−0.12] ***	−1.53 (0.27) [−0.07] ***	−1.53 (0.27) [−0.07] ***	−2.94 (0.30) [−0.14] ***	−2.88 (0.30) [−0.14] ***	−2.81 (0.30) [−0.13] ***
Two occurrences (N=499)	−5.84 (0.34) [−0.24] ***	−4.97 (0.33) [−0.20] ***	−4.80 (0.34) [−0.19] ***	−3.88 (0.39) [−0.14] ***	−1.95 (0.33) [−0.07]***	−1.88 (0.33) [−0.07]***	−5.30 (0.36) [−0.21] ***	−4.91 (0.36) [−0.19] ***	−4.85 (0.36) [−0.19) ***
Three occurrences (N=368)	−8.60 (0.39) [−0.30]***	−7.34 (0.38) [−0.26] ***	−7.04 (0.40) [−0.25] ***	−5.73 (0.45) [−0.18] ***	−3.08 (0.38) [−0.10] ***	−2.82 (0.39) [−0.09] ***	−7.55 (0.41) [−0.26] ***	−6.91(0.41) [−0.24] ***	−6.88 (0.43) [−0.24] ***

† Overall well-being (CASP-19).

‡Physical well-being (SF-36).

§Mental well-being (SF-36).

¶Difference (and SE) in well-being score from the reference group.

†† A test of trend analysis indicated a trend for each of the well-being outcomes across the occurrences of short sleep (CASP-19, p≤0.001; SF-36 (PCS), p≤0.001; SF-36 (MCS), p≤0.001).

‡‡ A test of trend for well-being outcomes over the occurrences of long sleep was only significant for physical well-being (SF-36 (PCS), p=0.011).

§§A test for trend showed a trend of each well-being outcome across the occurrence of insomnia symptoms, (CASP-19, p≤0.001; SF-36 (PCS), p≤0.001; SF-36 (MCS), p≤0.001). Figures in square brackets show the difference in standardised well-being scores from the reference group. Model 1: adjusted for age, age^2^, gender, wealth; model 2: adjusted as in model 1+employment status, marital status, limiting health conditions, physical functioning (ADL/IADL), health behaviours (alcohol, physical activity, smoking, BMI); model 3: adjusted as in model 2+insomnia symptoms/recurrent long or short sleep. ***p≤0.001, **p≤0.01, * p<0.0.

ADL, activities of daily living; BMI, body mass index; CASP-19, Control, Autonomy, Self-realisation and Pleasure measure; IADL, instrumental activities of daily living; SF-36 (MCS), Short Form Health Survey mental well-being component scores; SF-36 (PCS), Short Form Health Survey physical component scores.

A similar pattern of results were observed for mental well-being. However, for physical well-being the association between three reported occurrences of short sleep, although attenuated, remained in model 3. The results for reported recurrent long sleep (≥9 h) showed that one occurrence was associated with both lower overall and mental well-being, although this was attenuated by model 3 for overall well-being. However, for physical well-being there was a negative association between two or more occurrences of long sleep, which although attenuated, remained in each of the three models. A test of trend for well-being outcomes over the occurrences of long sleep was only significant for physical well-being (SF-36 (PCS), p=0.011).

Table 3 shows the association of the three sleep exposures with overall, physical and mental well-being after further adjustment for the potential confounding effects of depression. Model 3 (from table 2) is additionally adjusted for the modified GHQ-30 depressive symptom score. Overall, the pattern of findings observed previously remains consistent, although the size of the association is attenuated, especially for overall well-being. Online supplementary table S3 compares the key characteristics of those included and not included in the analyses. Although well-being scores and participant characteristics were similar between this sample and those excluded due to missing data; chronic insomnia symptoms and recurrent short sleep were more common and well-being poorer among those not included in the analyses.

**Table 3:** Association of recurrent sleep duration and insomnia symptoms with well-being after further adjustment for depressive symptoms†

N=4491	Overall well-being Diff‡ (SE) [standardised difference]	Physical well-being Diff‡ (SE) [standardised difference]	Mental well-being Diff‡ (SE) [standardised difference]
Recurrent short sleep			
No short sleep	0.00 REF	0.00 REF	0.00REF
One occurrence	−0.62 (0.35) [−0.02]	0.01 (0.37) [−0.00]	−0.12 (0.31) [−0.01]
Two occurrences	−0.72 (0.53) [−0.02]	−0.56 (0.57) [−0.01]	0.46 (0.48) [0.01]
Three occurrences	−0.55 (0.64) [−0.01]	−1.63 (0.68)** [−0.03]	0.70 (0.58) [0.01]
Recurrent long sleep			
No long sleep	0.00 REF	0.00 REF	0.00 REF
One occurrence	−0.94 (0.54) [−0.02]	−0.66 (0.58) [−0.01]	−1.25 (0.49)* [−0.03]
Two or three occurrences	−0.14 (0.84) [−0.00]	−3.47 (0.90) *** [−0.04]	−0.30 (0.76) [−0.00]
Chronic insomnia symptoms			
No insomnia symptoms	0.00 REF	0.00 REF	0.00 REF
One occurrence	−1.76 (0.26) *** [−0.09]	−1.36 (0.27)*** [−0.06]	−1.22 (0.23)*** [0.06]
Two occurrences	−3.28 (0.31)*** [−0.13]	−1.61 (0.33)*** [−0.06]	−2.31 (0.28)*** [−0.09]
Three occurrences	−4.84 (0.37)*** [−0.17]	−2.41 (0.40)*** [−0.08]	−3.22 (0.34)*** [−0.11]

†Estimates are adjusted as in model 3 (see tables 3 and 4) with additional adjustment for depressive symptoms score.

‡Difference (and SE) in well-being score from the reference group. Figures in square brackets show the difference in standardised well-being scores from the reference group. ***p≤0.001, **p≤0.01, * p<0.05.

## Discussion

Prospective repeat data over 10 years of follow-up suggest that insomnia symptoms and long sleep are independently associated with lower levels of well-being, measured as overall well-being, physical and mental well-being. There is a dose–response association between chronic insomnia symptoms and poorer well-being, independent of sleep duration and depressive symptoms. However, the association between sleep duration and well-being differed according to the measure of well-being examined, possibly an indication that as societies age, there may be less homogeneity in older age groups and the correlates of well-being at older age may vary.

Our findings agree with previous research, which has demonstrated independent negative associations, between insomnia symptoms and lower physical and mental well-being scores.^28–34^ We are not aware of any studies that have examined the association between chronic exposure to insomnia symptoms and the SF-36. We found a dose–response association, suggesting that recurrent exposure to insomnia was associated with both lower mental and physical well-being.

Previous cross-sectional work has shown an association between sleep duration and both mental and physical well-being.^10^ ^35^ We found that recurrent exposure to long or short sleep was associated with poorer physical well-being. However, we did not find a prospective association between sleep duration and mental well-being. The association between recurrent short sleep and mental well-being was no longer significant after insomnia symptoms were taken into account. However, recurrent short sleep in the absence of high levels of insomnia symptoms does not necessarily predict poor well-being. Faubel *et al*^10^ also found that sleep duration at baseline failed to predict change in mental well-being 2 years later.

Studies that have examined the relationship between both short and long sleep with overall well-being have generally reported an initial U-shaped relationship,^11^ ^12^ which did not always remain after adjustment.^12^ This did not accord with our cross-sectional findings, where only short sleep was related to well-being. Additionally, we did not find an association between recurrent short or long sleep and overall well-being. However, in accordance with others,^7–9^ ^12^ we found an independent association between chronic insomnia symptoms and lower overall well-being, which remained even when depressive symptoms were taken into account.

Many of the mechanisms suggested as explanations for the association between insomnia symptoms and well-being are similar to those suggested for short sleep,^11^ ^28^ implying that both indicators are simply capturing an underlying concept of poor quality sleep.^36^ ^37^ However, we find a dose–response association for insomnia symptoms and well-being which is not present for short sleep, suggesting that there may be different mechanisms for these associations.

A number of mechanisms may mediate the association between short sleep and overall or mental well-being, including fatigue or sleepiness during the day^38^ and the involvement of metabolic and endocrine functions.^39^ The mechanisms linking long sleep and physical well-being are less clear, possibilities are reverse causation, as longer sleep may be an early symptom of undiagnosed disease,^10^ or increased sleep fragmentation.^40^ ^41^ However, associations were robust to adjustment for presence of a limiting long-term illness. Associations between well-being and physical well-being may also be subject to confounding by mental health problems such as depression, where reporting problems with sleep is a clinical symptom.^42^ However, the association between sleep duration and insomnia symptoms remained following adjustment for the GHQ depression scale.

We used self-reported measures of both sleep duration and insomnia symptoms. Observational studies are beginning to include measures of sleep duration based on actigraphy data; however, these were not available in 1997, when sleep duration was first measured in this cohort. Also as sleep problems remain self-diagnosed within the primary care setting, self-reported data can be assumed to have face validity. Self-reported sleep duration has shown moderate correlations with more objective measures of sleep, such as actigraphy.^43–45^ Despite this, further research will be necessary when long-term actigraphy measures of sleep are available, since three measurements in 10 years may not fully describe the sleep history of participants. Second, we are not able to take sleep disorders such as sleep apnoea into account. However, controlling for BMI in our analysis should reduce potential confounding by sleep apnoea, since the prevalence of obesity is greater in those with this sleep condition. There is a potential overlap between the measures of vitality included in the SF-36 scale and the Jenkins questionnaire which asks respondents about waking up feeling ‘tired and worn out’. A sensitivity analysis was undertaken in the cross-sectional analysis to examine any potential overlap between these questions and it was found that removing them had little effect on the results. The participants in Whitehall II were originally from an occupational cohort of white-collar workers, and therefore participants were employed and relatively healthy; this may limit generalisability. Further caution should also be exercised extrapolating these conclusions to a general population, due to drop-outs from the sample originally enrolled in the study. The strengths of this work are the availability of three repeat measures of exposure to short or long sleep and insomnia symptoms and three validated well-being outcomes for a large sample of participants from a well-characterised cohort. We conclude that while chronic insomnia symptoms are negatively associated with all aspects of well-being. However, for older adults, recurrent short sleep duration does not necessarily have a negative effect on overall or mental well-being, when the effects of insomnia symptoms are taken into account. However, extreme sleep duration is associated with poor physical well-being.
